# Epidemiology of early-onset and late-onset invasive infections in Australian neonates and infants: a retrospective multicentre study

**DOI:** 10.1136/bmjph-2025-002733

**Published:** 2025-10-13

**Authors:** Phoebe C M Williams, Mona Mostaghim, Jackson Harrison, Monica Lahra, Mark Greenhalgh, Matthew O’Sullivan, Michael Maley, Ju-Lee Oei, Archana Koirala, Himanshu Popat, Kei Lui, Brendan McMullan, Pamela Palasanthiran, Alison Kesson, David Isaacs, Adam William Bartlett

**Affiliations:** 1 Sydney Institute for Infectious Diseases, School of Public Health, Faculty of Medicine, The University of Sydney, Sydney, New South Wales, Australia; 2 School of Women & Children’s Health, University of New South Wales, Sydney, New South Wales, Australia; 3 Department of Paediatric Infectious Diseases, Sydney Children’s Hospital Randwick, Randwick, New South Wales, Australia; 4 Department of Microbiology, NSW Health Pathology, Sydney, New South Wales, Australia; 5 Department of Neonatology, Royal Prince Alfred Hospital, Camperdown, New South Wales, Australia; 6 Centre for Infectious Diseases and Microbiology, NSW Health Pathology, Sydney, New South Wales, Australia; 7 Department of Microbiology & Infectious Diseases, NSW Health Pathology, Liverpool, New South Wales, Australia; 8 Department of Neonatology, The Royal Hospital for Women, Randwick, New South Wales, Australia; 9 Department of Infectious Diseases, Nepean Hospital, Penrith, New South Wales, Australia; 10 Department of Neonatology, The Children’s Hospital at Westmead, Westmead, New South Wales, Australia; 11 NHMRC Clinical Trial Centre, University of Sydney, Sydney, New South Wales, Australia; 12 Newborn Care, Australian and New Zealand Neonatal Network, Royal Hospital for Women, National Perinatal Epidemiology and Statistic Unit, University of NSW, Sydney, New South Wales, Australia; 13 Department of Infectious Diseases and MIcrobiology, Sydney Children’s Hospital, Westmead, New South Wales, Australia; 14 Paediatric Infectious Diseases, The Kirby Institute, University of NSW, Sydney, New South Wales, Australia

**Keywords:** Epidemiology, Epidemics, Disease Transmission, Infectious, Age Factors

## Abstract

**Background:**

There has been little decline in neonatal mortality rates over recent decades, and this is now further challenged by the rising prevalence of antimicrobial resistance. In Australia, the incidence of neonatal sepsis is low on a global scale, yet there are increasingly frequent outbreaks of multidrug-resistant (MDR) infections in neonatal intensive care units, alongside rising rates of colonisation with MDR bacteria.

**Methods:**

We analysed positive blood and cerebrospinal fluid cultures collected from infants (aged 0 to ≤180 days) across five clinical sites in Australia between 2010 and 2019, to determine evolving antimicrobial susceptibility profiles.

**Results:**

After excluding presumed contaminants, we analysed 743 pathogenic bacterial isolates cultured from 624 neonates and infants with early-onset (≤72 hours), late-onset (>72 hours to ≤28 days) and very late-onset (>28 days to ≤180 days) infections. *Escherichia coli* (37%) and *Streptococcus agalactiae* (31%) were the primary pathogens responsible for early-onset bloodstream infections, while coagulase-negative staphylococci, *E. coli* and *Staphylococcus aureus* were responsible for most infections in older neonates and infants. Antimicrobial susceptibility to currently recommended empiric regimens remains high; however, gram-negative bacteria—including MDR bacteria—were responsible for an increasing proportion of very late-onset infections over the study period (22% in 2010–2014 vs 34% in 2015–2019; p=0.07).

**Conclusions:**

Although empiric antimicrobial regimens remain adequate for most pathogens causing infections in neonates and infants in Australia, there is an increasing burden of invasive infections caused by gram-negative bacteria. Ongoing surveillance is necessary to ensure empiric antimicrobial guidelines remain efficacious and appropriate.

**WHAT IS ALREADY KNOWN ON THIS TOPIC**

- Neonatal sepsis is a major cause of child mortality. As antimicrobial resistance (AMR) increases globally, the incidence of multidrug-resistant neonatal infections is rising. There is evidence to suggest a shift in the epidemiology of pathogens traditionally attributed to be causative of early-onset and late-onset neonatal infections is occurring, as increasingly premature babies require prolonged hospitalisation and may be vulnerable to infections with multidrug-resistant nosocomial pathogens. As AMR rises, it is important to monitor the pathogens causative of serious bacterial infections in neonates and infants, to ensure empirical regimens recommended to treat sepsis and meningitis remain appropriate.

**WHAT THIS STUDY ADDS**

- We found high rates of susceptibility to empiric antibiotic regimens recommended to treat early-onset and late-onset infections in neonates and young infants in Australia. However, there was a rising burden of invasive infections caused by gram-negative bacteria—including multidrug-resistant pathogens—evident in infants (29–180 days) over the later years of our one-decade study period. *Escherichia coli* was the most frequent cause of bloodstream infections identified across neonates and infants, though *Enterobacter* spp and *Klebsiella* spp were responsible for an increasing proportion of very late-onset bloodstream infections. *Streptococcus agalactiae* was the most frequently isolated bacteria causative of meningitis in neonates and young infants.

**HOW THIS STUDY MIGHT AFFECT RESEARCH, PRACTICE OR POLICY**

- In Australia’s (high-income) healthcare setting, susceptibility to currently recommended antibiotic regimens for treating serious bacterial neonatal infections remains high. However, our study—unique in its inclusion of infants up to 180 days of age—has revealed a significant burden of gram-negative bacterial infections that occur beyond the neonatal period. As increasingly premature neonates are successfully resuscitated globally, requiring prolonged hospitalisation for their survival, strategies to reduce their risk of colonisation and infection with gram-negative bacteria require focused research effort—particularly in the context of rising AMR.

## Introduction

 Over the past three decades, there has been global progress in reducing child mortality, yet there has been a slower decline in newborn mortality—with nearly half of all childhood deaths now occurring within the neonatal (0–28 days) period.^1^ Serious bacterial infections (often referred to as ‘neonatal sepsis’) account for up to 700 000 newborn deaths each year—the majority of which occur in Southeast Asia and sub-Saharan Africa.^2 3^ However, as antimicrobial resistance (AMR) rises globally, the mortality and disability burden caused by multidrug-resistant organisms (MROs)—which are increasingly responsible for a larger proportion of neonatal infections—requires urgent attention across all healthcare settings.^3^

The newborn period bears the highest lifetime risk of sepsis—a clinical syndrome that may be caused by viral, fungal or bacterial pathogens.^3^ Multiple risk factors predispose neonates (and infants, particularly those born prematurely) to invasive bacterial infections, which are the most common cause of neonatal sepsis—including immune system immaturity, poorly developed gastrointestinal and skin mucosal barriers, and exposure to invasive procedures when unwell.^4^ In Australia, the incidence of neonatal sepsis is low on an international scale yet is gradually increasing,^5^ and the associated morbidity and mortality burden is significant.^6^

Advanced healthcare systems now enable the resuscitation of neonates from as early as 22 weeks’ gestation, and these extremely preterm neonates subsequently require a prolonged hospital stay during which they are at high risk of developing late-onset sepsis (LOS) (with incidence rates up to 15 times that observed term infants).^7 8^ Furthermore, premature infants are frequently prescribed multiple empiric antibiotic courses, reducing their microbiome diversity and increasing the risk of colonisation and infection with MROs.^9^

International observational studies have revealed the epidemiology of neonatal sepsis is rapidly evolving due to the growing global burden of AMR.^10^,^12^ However, there are scarce published data delineating the epidemiology of neonatal infections in Australian infants, with most published literature focussing on early-onset sepsis (EOS; currently defined as infections occurring at ≤72 hours of age).^6 8^ While EOS is an important cause of neonatal morbidity and mortality, understanding the burden of LOS (occurring at >72 hours of age) is increasingly important as more premature infants requiring prolonged hospitalisation survive.^13^

In Australia and New Zealand, LOS occurs in 44% of neonates born at <24 weeks’ gestational admitted to tertiary neonatal intensive care units (NICUs).^14^ While LOS can occur secondary to perinatally acquired (vertically transmitted) pathogens, it is often the consequence of acquisition of MROs via colonisation with bacteria present within the hospital setting.^15^ However, there is increasing evidence to suggest hospital-acquired bacteria may also be responsible for a significant burden of EOS, challenging the traditional rationale behind the empirical antibiotic regimens currently recommended to treat EOS and LOS, which are based on historical data around causative bacteria.

Australia’s therapeutic guidelines recommend benzylpenicillin plus gentamicin as empiric antibiotics to treat EOS (or benzylpenicillin plus cefotaxime if meningitis is suspected),^16^ and an aminopenicillin plus gentamicin for infants with community-acquired LOS (cefotaxime where meningitis is suspected), with the addition of vancomycin if there is an epidemiological risk factor for methicillin-resistant *Staphylococcus aureus*. For infants aged >2 months with septic shock or meningitis, these guidelines recommend gentamicin, cefotaxime/ceftriaxone and vancomycin for infants presenting from the community.^16^ Most hospital-based and state-based guidelines across Australia recommend neonates (and infants hospitalised since birth) with LOS are treated with flucloxacillin and gentamicin, ±vancomycin.^16^,^19^

Despite strong antimicrobial stewardship programmes across Australia’s healthcare settings, there are emerging challenges with both neonatal MRO colonisation and infection outbreaks in NICUs,^1320^,^22^ which are increasingly difficult to treat.^23 24^ As the burden of AMR evolves and neonates are resuscitated at earlier gestations, significant knowledge gaps remain in understanding the epidemiology of serious bacterial infections in neonates and infants—including evaluation as to whether the current empiric antibiotic recommendations remain appropriate.^25 26^ We aimed to address this evidence gap by analysing the contemporary bacterial pathogens (and their evolving antibiotic susceptibility profiles) responsible for both early-onset and late-onset serious bacterial infant infections in New South Wales (NSW), Australia’s most populous state.

## Methods

We conducted a multicentre retrospective observational study across four tertiary urban hospitals and one rural referral hospital in NSW, Australia. Laboratory databases were systematically interrogated to identify positive blood and cerebrospinal fluid (CSF) cultures collected between 2010 and 2019 (inclusive) in infants aged ≤180 postnatal days of age. We stratified our analysis of culture-positive infections to (1) the early neonatal period (≤72 hours), (2) the late neonatal period (>72 hours to ≤28 days) and (3) very late-onset infections (>28 days to ≤180 days). State-wide data were used to ascertain denominator data for the number of livebirths within each of the study hospitals.^20^

Cultures collected within 14 days of an index culture isolating the same pathogen were considered duplicates and removed, alongside a predefined list of likely contaminant bacteria (online supplemental figure 1). Coagulase-negative staphylococci (CoNS) isolates were only retained as bloodstream infection pathogens in infants aged >72 hours of age who were admitted to the neonatal intensive care, or in CSF isolates where review of clinical data confirmed the occurrence of meningitis. CoNS isolates from neonates admitted from the community or in postnatal wards were removed as presumed contaminants.

Antimicrobial susceptibility data were attained following testing performed using automated methods (Vitek2, BioMérieux or BD Phoenix, Becton Dickinson, USA) and categorised by Clinical and Laboratory Standards Institute (CLSI),^27^ European Committee on Antimicrobial Susceptibility Testing (EUCAST)^28^ or Calibrated Dichotomous Sensitivity test (CDS) methodology^29^; then extracted from laboratory systems and evaluated against a predefined list of antibiotics (online supplemental table 1). Pathogens with (non-inherent) non-susceptibility to ≧3 classes of antibiotics were classified as multidrug-resistant (MDR). Changes in causative bacteria and antimicrobial susceptibility were assessed using χ^2^ tests, to compare the periods between January 2010–December 2014 and January 2015–December 2019.

Data cleaning and statistical analysis were performed in the R programming environment (R Core Team, 2023). Continuous data were reported as median and IQRs, while categorical variables were reported as numbers and percentages, and compared using χ^2^ or Fisher’s exact tests. Poisson regression was used to assess trends in rates of EOS across the study period.

### Patient and public involvement

The research question was developed by the authorship group which includes parents with lived experience as carers of hospitalised infants suffering from neonatal sepsis. The patients whose data were included in this study were not involved in the study design or analysis (nor were their carers), as a waiver of consent was provided via the ethical committee, due to this analysis being conducted as a retrospective, anonymised review of laboratory databases.

## Results

**Figure 1:**
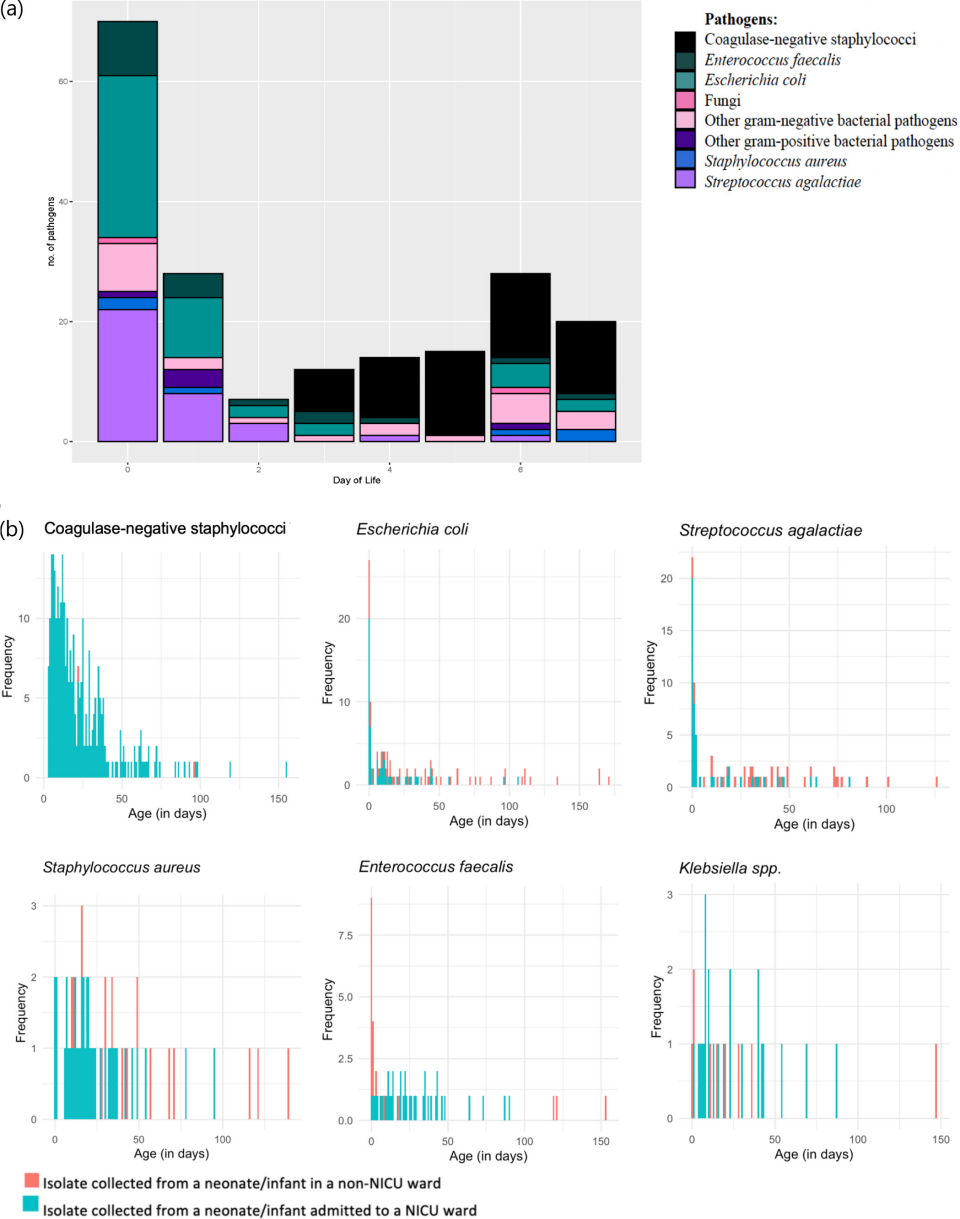
Incidence of common pathogens causing infections in neonates and infants by day of postnatal age; across days 0–7 (**a**); and days 0–180 (**b**). **(a**) Pathogens responsible for invasive infections in neonates over the first week of life. *Organisms included in ‘other’ gram-negative: *Acinetobacter* spp, *Burkholderia cepacia, Citrobacter freundii, Enterobacter* spp, *Klebsiella* spp, *Morganella* morganii, *Serratia marcescens, Pseudomonas* spp and ‘other’ gram-positive: *Streptococcus anginosus, Streptococcus pneumoniae, Streptococcus pyogenes.* (**b**) Incidence of common pathogens causative of invasive infections in neonates and young infants across days 0–180. NB: Note the variation in the y-axis based on pathogen prevalence. Two CoNS infections reported in infants admitted to postnatal wards were from clinically confirmed CNS infections. CoNS, coagulase-negative staphylococci; NICU, neonatal intensive care unit.

We analysed 743 pathogenic bacterial isolates collected from 624 infants across 5 hospitals during the study period (figure 1, table 1, online supplemental figure 1). This included 464 isolates from neonates and infants admitted to Nepean, Westmead and Wagga Wagga Base Hospital between 2010 and 2019 (inclusive); and 279 isolates from neonates and infants admitted to The Royal Hospital for Women and Sydney Children’s Hospital from 2015 to 2019 (a shorter period than the predefined study period, due to laboratory information system changes).

**Table 1:** Demographic characteristics

	Total	0–72 hours	>72 hours to 27 days	28–180 days
Number of infection episodes	**705**	106	365	234
Patients	**624**	103	342	211
Gender				
Female	**291** (**41**)	41 (39)	152 (42)	98 (42)
Male	**406** (**58**)	57 (54)	213 (58)	136 (58)
Unknown	**8 (1**)	8 (7.5)	0	0
Age (hours/days)	**16 (7–35**)	0 (0–1)	12 (8–18)	45 (35–70.5)
Admitted to the neonatal intensive care unit at time of positive culture	**543** (**77**)	75 (71)	321 (88)	147 (63)
Time period				
2010–2014	**252** (**36**)	38 (36)	140 (38)	74 (32)
2015–2019	**453** (**64**)	68 (64)	225 (62)	160 (68)
Specimen type				
Bloodstream	**669** (**95**)	101 (95)	353 (97)	215 (92)
Cerebrospinal fluid	**36** (**5**)	5 (5)	12 (3)	19 (8)
Polymicrobial infections*				
Bloodstream	**34** (**5**)	4 (4)	17(5)	13 (6)
Cerebrospinal fluid	**1 (<1**)	0	0	1 (5)
Hospital				
Nepean Hospital	**137** (**19**)	30 (28)	70 (19)	37 (16)
Royal Hospital for Women	**165** (**23**)	17 (16)	96 (26)	52 (22)
Sydney Children’s Hospital	**98** (**14**)	8 (8)	27 (7)	63 (27)
Wagga Wagga Base Hospital	**18** (**3**)	8 (8)	7 (2)	8 (3)
The Children’s Hospital at Westmead	**287** (**41**)	48 (45)	165 (45)	74 (32)

Data are n (%) or median (IQR).

* % of specimen type.

The incidence of EOS across the study period varied from 0.6 per 1000 livebirths to 1 per 1000 livebirths (online supplemental table 2), with no significant trend in EOS incidence rates observed across the study period (0.05% change in incidence per year, 95% CI −7.1% to 8.9%).

**Figure 2:**
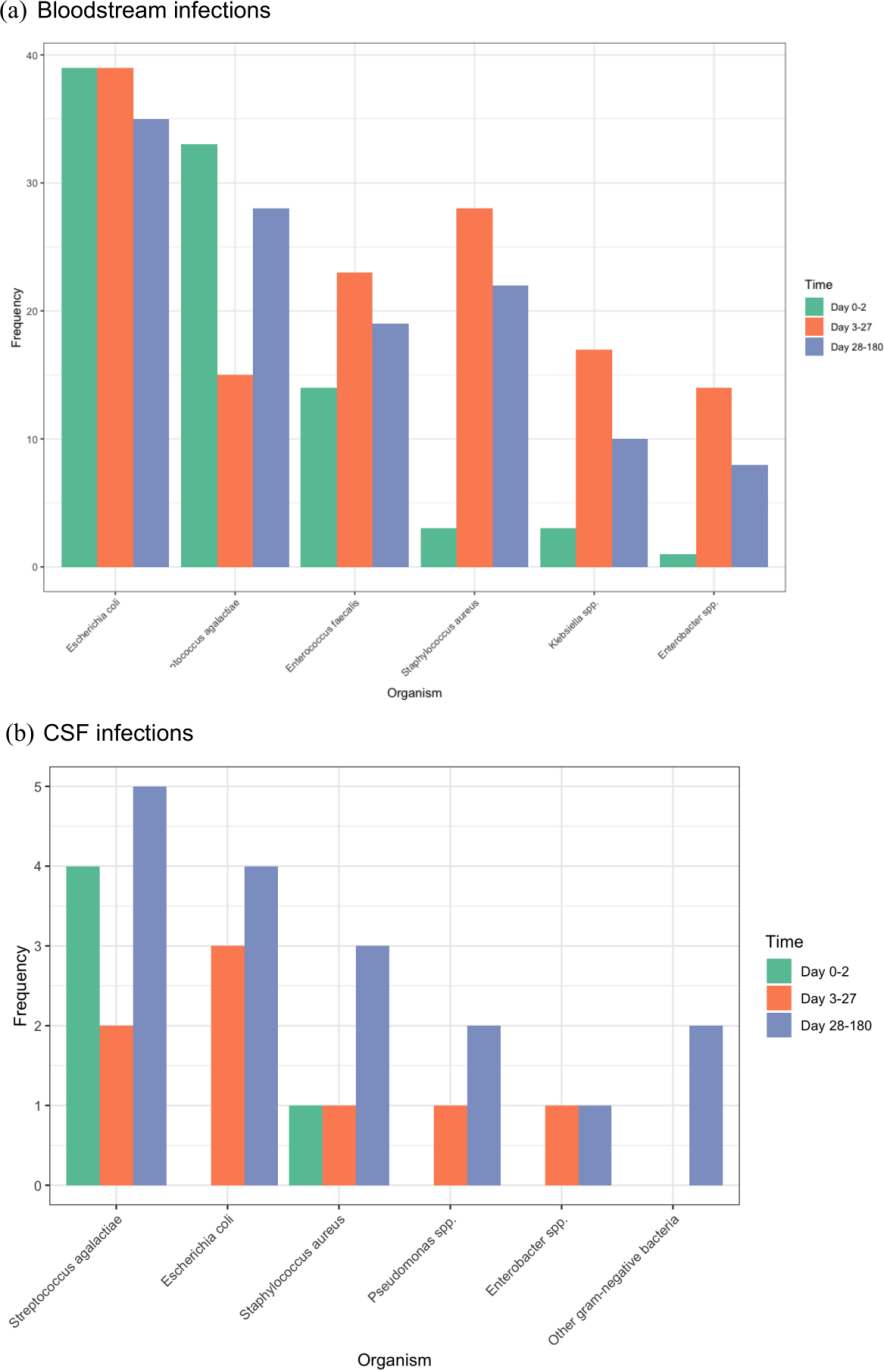
Most frequently identified pathogens causative of (**a**) bloodstream and (**b**) CSF infections in neonates and infants. CSF, cerebrospinal fluid.

Bloodstream infections were responsible for most infection episodes (95%, 669/743), with 35 CSF infections also identified (of 743, 5%). There were 34 polymicrobial bloodstream infections (≥2 pathogenic bacteria isolated), and one CSF specimen yielded three pathogenic bacteria (table 1, figure 2). Most isolates were obtained from neonates or infants admitted to NICUs (73%, 543/743).

### Early-onset neonatal infections (≤72 hours of age; days 0–2 of life)

15% (106/705) of infections occurred in the EOS period (≤72 hours of age), with a median age of infection at 0 days (IQR 0–1 day). Bloodstream infections were caused by approximately equal proportions of gram-negative (48%, 50/105) and gram-positive (51%, 54/105) bacteria, with one episode of fungaemia (due to *Candida albicans*) (table 2, figure 1). The proportion of gram-negative bacteria responsible for EOS was similar across the study period (58% over 2010–2014 vs 43% over 2015–2019, p=0.13).

**Table 2:** Pathogens causative of bloodstream and CSF infections in neonates and infants 0–180 days of age

	Blood			CSF			Total
	0–72 hours	>72 hours to 27 days	28–180 days	0–72 hours	>72 hours to 27 days	28–180 days	
	n=105	n=370	n=230	n=5	n=12	n=21	
Gram-negative bacteria	50 (48)	91(25)	68 (30)	0 (n/a)	5 (42)	11 (52)	225
*Escherichia coli*	39 (37)	39 (10)	35 (15)	0	3 (25)	4 (19)	120
*Klebsiella* spp	3 (3)	17 (5)	10 (4)	0	0	1 (5)	31
*Pseudomonas* spp	2 (2)	10 (3)	4 (2)	0	1 (8)	2 (9.5)	19
*Enterobacter* spp	1 (1)	14 (4)	8 (3)	0	1 (8)	1 (5)	25
*Serratia marcescens*	1 (1)	3 (1)	4 (2)	0	0	1 (5)	9
*Acinetobacter* spp	1 (1)	6 (2)	4 (2)	0	0	0	11
Other gram-negative bacteria	3 (3)	2 (0.5)	3 (1)	0	0	2 (10)	10

Gram-positive bacteria	54 (51)	278 (75)	162 (70)	5 (100)	7 (58)	10 (48)	516
Coagulase negative staphylococcus*	n/a	209 (56)	88 (38)	0	3 (25)	2 (10)	302
*Enterococcus faecalis*	14(13)	23 (6)	19 (8)	0	1 (8)	n/a	57
*Streptococcus agalactiae*	33(31)	15 (4)	28 (12)	4 (80)	2 (17)	5 (24)	87
*Staphylococcus aureus*	3 (3)	28 (8)	22 (10)	1 (20)	1 (8)	3 (14)	58
Other gram-positive bacteria	4 (4)	3 (1)	5 (2)	0	0	0	12

Fungal	1 (1)	1 (<1)	0	0	0	0	2
*Candida albicans*	1 (1)	1 (<1)	0	0	0	0	2

Data are n (%).

* Coagulase negative staphylococci excluded when reported on general wards or days 0–2 of life.

CSF, cerebrospinal fluid; n/a, not available.

*Escherichia coli* (37%, 39/105), *Streptococcus agalactiae* (31%, 33/105) and *Enterococcus faecalis* (13%, 14/105) were identified as the bacteria most frequently responsible for EOS (figure 1). Inborn neonates who required admission to the NICU for higher-level care (due to prematurity, prenatal or perinatal complications) were more likely to have *S. agalactiae* infections (41%, vs 12% of infants admitted to postnatal wards), while neonates with *E. faecalis* infections were more likely to be admitted to the postnatal ward (32% vs 4% of infants admitted to the NICU). All CSF infections (n=5) in this age group were caused by gram-positive bacteria (*S. agalactiae* and *S. aureus*) (table 2).

### Late-onset neonatal infections (>72 hours to <28 days)

Most of the culture-positive infections identified in our study (52%, 365/705) occurred in the late-onset neonatal period (>72 hours to <28 days), at a median age of 12 days (IQR 8–18). Within this cohort, there were 353 episodes of bacteraemia (incorporating 370 isolates, including 17 polymicrobial infections) and 12 CSF infections (caused by *E. coli, S. agalactiae, S. aureus, Pseudomonas aeruginosa, Enterobacter* spp and CoNS; table 2).

Gram-positive bacteria predominated as causative of bloodstream infections (n=278, 75%), largely due to a significant burden of CoNS isolated from neonates admitted to the NICU (209/370, 56%), followed by *S. aureus* (8%, 28/370), *E. faecalis* (6%, 23/370) and *S. agalactiae* (4%, 15/370). One episode of fungaemia was reported (*C. albicans*).

The most frequently isolated gram-negative bacteria causative of late-onset neonatal bloodstream infections was *E. coli* (10%, 39/370), followed by *Klebsiella* spp (5%, 17/370) and *Enterobacter cloacae* complex (14/370, 4%). The proportion of bloodstream infections caused by gram-negative bacteria was largely consistent across the two periods (21% in 2010–2014 and 27% in 2015–2019, p=0.26).

### Very late-onset infections in infants (days 28–180 inclusive)

One-third of isolates (234/705, 33%) were cultured from infants aged 28–180 days (median age of infection: 45 days, IQR 35–71). Among the pathogens causative of 21 CSF infections, *S. agalactiae* (5/21), *E coli* (4/21, 19%) and *S. aureus* (3/21, 14%) were isolated.

Gram-positive bacteria predominated as causative of bloodstream infections in this age group (162/230, 70%), once again due to a significant burden of CoNS in NICU-admitted infants (38%, 88/230), followed by very late-onset *S. agalactiae* infections (12%, 28/230), *S. aureus* (10% 22/230) and *E. faecalis* (8%, 19/230). Among gram-negative bacteria, *E. coli* was responsible for 15% of all bloodstream infections (35/230) followed by *Klebsiella* spp (10/230, 4%) and *Enterobacter* spp (8/230, 3%). Across all hospitals, there was a trend towards gram-negative bacteria causing more very late-onset bloodstream infections in the latter part of the study period (22% over 2010–2014 vs 34% over 2015–2019; p=0.07).

### Antimicrobial susceptibility profiles

#### Gram-positive bloodstream infections

*S. agalactiae* was a prominent cause of bloodstream infections (n=76 isolates), with all tested isolates susceptible to penicillins and third-generation cephalosporins (cefotaxime/ceftriaxone) and most susceptible to clindamycin (27/39, 70%) and erythromycin (32/44, 73%). Among *S. aureus* isolates, 83% (44/53) were methicillin-susceptible (online supplemental table 3). There were insufficient and inconsistent antimicrobial susceptibility data available to enable evaluation of the CoNS bacteria isolated.

#### Gram-negative bloodstream infections

*E. coli* was the primary cause of gram-negative bloodstream infections in our study population (n=113; figure 2, online supplemental table 4). Of the isolates tested, all were susceptible to amikacin (75/75) and meropenem (78/78), 92% (100/109) demonstrated susceptibility to third-generation cephalosporins, 89% (101/113) were gentamicin/tobramycin susceptible, and only 45% were aminopenicillin susceptible (33/74). Trimethoprim-sulfamethoxazole susceptibility decreased for *E. coli* isolates over the study period (from 85% (33/39) in 2010–2014 to 65% (22/34) in 2015–2019, p=0.05), yet no other statistically significant susceptibility changes were observed.

There were 14 episodes of MDR *E. coli* bacteraemia (defined as acquired non-susceptibility to ≧1 antibiotic across ≧3 antimicrobial classes), including eight (57%) which were extended-spectrum beta-lactamase producing pathogens. Most of these infections (9/14) occurred at Nepean and Westmead hospitals over the latter part of the study period (2015–2019 vs 5/14 in 2010–2014, p=0.18)

*E. cloacae* complex was the second most common gram-negative pathogen responsible for bloodstream infections (n=23). Most *E. cloacae* complex isolates tested were susceptible to meropenem (95%, 19/20), cefepime (14/16, 88%), gentamicin/tobramycin (87%), amikacin (16/16, 100%), ciprofloxacin (19/19) and trimethoprim-sulfamethoxazole (17/17). Two MDR *E. cloacae* infections (including one carbapenem-resistant infection) were from infants admitted to Royal Hospital for Women & Sydney Children’s Hospital (in 2017 and 2019).

#### Antimicrobial susceptibility in CSF infections

Gram-negative CSF infections were primarily caused by *E. coli* (7/16. 44%)*,* with all tested CSF isolates (n=7) universally susceptible to third-generation cephalosporins (ceftriaxone/cefotaxime) and aminoglycosides (online supplemental table 5). *E. cloacae* complex CSF isolates (n=2) and *Pseudomonas aeruginosa* CSF isolates (n=2) were susceptible to aminoglycosides (gentamicin/tobramycin) and later-generation cephalosporins. Gram-positive CSF infections were primarily caused by *S. agalactiae*, with all isolates susceptible to penicillin (11/11) and ceftriaxone (5/5). One *S. aureus* CSF isolate was methicillin-resistant (of 5, 20%; online supplemental table 6).

#### Antimicrobial susceptibility to commonly prescribed antibiotics

Table 3 summarises the susceptibility of the most frequently isolated pathogens to commonly prescribed empiric antimicrobials. *E coli* isolates from infants with very late-onset infections were less likely to be susceptible to ampicillin than those attained from neonates, though gentamicin and third-generation susceptibility remained high across all age cohorts. Methicillin-resistant *S. aureus* evolved with increasing postnatal age, with no tested isolates exhibiting methicillin-resistance in the EOS period, while 34% and 13% of isolates revealed methicillin-resistance in older neonates (>72 hours to 27 days) and infants (days 28–180), respectively). *E. faecalis* isolates were all susceptible to vancomycin for the 0–2 days cohort, yet declined to 83% in older neonates (>72 hours to 27 days) and 89% in infants (28–180 days).

**Table 3:** Susceptibility profiles of the five most common pathogens causative of invasive (bloodstream and CSF) infections

	Total number of isolates	Ampicillin (isolates tested, % susceptible)	Gentamicin (isolates tested, % susceptible)	Flucloxacillin (isolates tested, % susceptible)	Vancomycin (isolates tested, % susceptible)	Ceftriaxone/ Cefotaxime (isolates tested, % susceptible)
0–72 hours (inclusive)						
*Escherichia coli*	39	32 (41%)	39 (82%)	N/A	2 (100%)	38 (89%)
*Streptococcus agalactiae*	37	N/A	NA	N/A	N/A	3 (100%)
*Enterococcus faecalis*	14	11 (91%)	2 (100)	N/A	13 (100%)	N/A
*Staphylococcus aureus*	4	N/A	N/A	4 (100)	4 (100%)	N/A
*Klebsiella* spp	3	1 (0%)	3 (67%)	N/A	N/A	3 (67%)

>72 hours to day 27 (inclusive)						
Coagulase negative staphylococcus	212	1 (100%)	1 (100%)	192 (8%)	185 (96%)	1 (100%)
*Escherichia coli*	42	28 (50%)	41 (90%)	1 (100%)	1 (100%)	39 (87%)
*Staphylococcus aureus*	29	1 (100%)	N/A	29 (66%)	23 (83%)	N/A
*Enterococcus faecalis*	24	16 (94%)	3 (100%)	4 (25%)	24 (83%)	N/A
*Klebsiella spp*	17	N/A	3 (100%)	N/A	1 (100%)	5 (100%)

Days 28–180 (inclusive)						
Coagulase negative staphylococcus	90	84 (0%)	84 (0%)	82 (6%)	78 (95%)	84 (0%)
*Escherichia coli*	39	27 (4%)	38 (8%)	39 (0%)	1 (100%)	38 (92%)
*Streptococcus agalactiae*	33	33 (0%)	33 (0%)	33 (0%)	1 (100%)	33 (30%)
*Staphylococcus aureus*	25	25 (0%)	25 (0%)	23 (87%)	15 (87%)	25 (0%)
*Enterococcus faecalis*	19	19 (5%)	18 (0%)	19 (0%)	19 (90%)	N/A

Summarised according to commonly recommended antimicrobial combinations, by age cohort.

CSF, cerebrospinal fluid; N/A, not available.

## Discussion

Our multicentre study evaluated the pathogens responsible for serious bacterial infections in neonates and infants (up to 6 months’ postnatal age) across five hospitals in urban and rural NSW, Australia. Our study revealed a significant burden of serious bacterial infections occur beyond the neonatal period, and that many of these infections are due to gram-negative pathogens, with a rising prevalence of multidrug-resistance evident over time. As healthcare settings are increasingly able to resuscitate and support extremely premature infants, prolonged hospital stay beyond the neonatal period necessitates the close monitoring of the epidemiology of these infections, particularly as the burden of AMR rises globally.

Our analysis is unique by way of inclusion of serious infections occurring beyond the neonatal period. Most published data in this field focuses on the epidemiology of early-onset neonatal infections, yet our study has revealed a substantial burden of serious bacterial infections occurring in infants. Late-onset bloodstream infections accounted for over half of all infections in our cohort, with a substantial proportion caused by CoNS in those admitted to NICUs. This finding aligns with published literature highlighting the significant role of CoNS in NICU-acquired infections, particularly in premature infants requiring long lines and prolonged hospitalisation.^30^ Many of these infants are prematurely born and vulnerable to hospital-acquired infections, emphasising the need for vigilant infection prevention and control (IPC) measures to mitigate nosocomial transmission of both CoNS and gram-negative bacteria (particularly given the propensity of gram-negative pathogens to acquire resistance mechanisms).^30^

Our data reveal adequate levels of susceptibility to currently recommended and commonly prescribed empiric antimicrobial combinations, suggesting local empiric antibiotic guidelines for sepsis and meningitis remain appropriate, aligning with other studies from high-income settings.^31 32^ However, MDR gram-negative infections were more prevalent in the latter part of our study period, and invasive bacterial infections occurring beyond the EOS period were more likely to exhibit antibiotic resistance. Ongoing evaluation of the appropriateness of empiric antibiotic regimens is increasingly necessary as the burden of AMR increases, to ensure these remain targeted to the most likely causative pathogens. This is particularly crucial in the context of the known challenges in confirming microbiological diagnoses in infants with invasive infections, which limits the capacity for directed therapy.^33^

Our study revealed interesting findings regarding the bacteria responsible for serious bacterial infections in neonates and infants admitted to various ward settings, with those admitted to higher-dependency settings (ie, NICUs) more likely to have infections with gram-negative pathogens (*E. coli*), while infants admitted to paediatric or postnatal wards at the time of their culture collection were more likely to have gram-positive infections (particularly *Enterococcus* spp). Whether this reflects differences in clinical acuity associated with pathogenic bacterial virulence is uncertain, and exemplifies the need for detailed prospective observational studies that incorporate both microbiological *and* clinical data. Indeed, the primary limitation of our study is the lack of clinical data available—including signs and symptoms evident at time of invasive isolate collection, risk factors for infection (such as the presence of a long line), and clinical outcomes—which poses a common challenge noted in AMR surveillance literature, and highlights the need for prospective surveillance studies that capture both microbiological and clinical data.^34^ Another limitation in our study is the change in laboratory information systems that occurred across two hospital sites during our study period, limiting available data to a shorter timeframe, and resulting in inconsistent availability of susceptibility data for some bacteria (particularly CoNS). Challenges in integrating laboratory information systems to easily procure data to examine epidemiological trends in infections is well-recognised AMR surveillance issue, and strategies to address this have been developed—including for resource-constrained healthcare settings, where the burden of neonatal infection is greatest.^35^

Despite these limitations, to our knowledge, this study is the most comprehensive analysis of the pathogens responsible for neonatal and infant bloodstream and CSF infections in Australia. We have revealed a low yet notable burden of serious bacterial infections caused by antibiotic-resistant bacteria, which will require close monitoring—particularly in the context of the very high rates of MDR gram-negative bacteria causing a significant neonatal sepsis burden in Australia’s neighbouring Southeast Asia region^10^,^1236 37^ Furthermore, our analysis of the pathogens causing serious bacterial infections by day of life underscores the importance of continued surveillance of the bacteria presumed to be responsible for EOS, both in Australia and globally.^38^Our study also revealed a very low burden of invasive fungal infections in neonates and infants, affirming the importance (and success) of fungal prophylaxis programmes across NICU settings in Australia, whereby infants with risk factors for invasive fungal infections (defined by local hospital policy, with guidance from the national neonatal formulary group)^39^ receive oral nystatin or fluconazole to prevent the risk of invasive fungal disease.^40^

Ongoing surveillance of the epidemiology of neonatal infections is clearly necessary as more babies are born and survive prematurely, and as AMR increases globally.^36 37^ This should incorporate wards across all geographic regions, and well-resourced and resource-constrained healthcare settings. Furthermore, studies to identify and evaluate targeted IPC interventions to reduce late-onset infections are also needed, to reduce the burden of nosocomial infections. Concurrently, evaluation of the transmission dynamics of MDR pathogens within NICUs—and how colonisation and invasive infection may interact—is necessary, to ensure IPC interventions can interrupt transmission chains. Finally, interventional studies to identify novel strategies (such as the role of steroids in neonatal meningitis, or the use of probiotics to protect the infant microbiome) to reduce the morbidity and mortality of neonatal infections are needed, alongside streamlined licensing of novel antibiotics to ensure neonates with MDR infections can access efficacious therapies.^23^ Together, these strategies may reduce the currently unacceptable mortality burden caused by neonatal sepsis globally.

## Supplementary material

10.1136/bmjph-2025-002733 online supplemental file 1

## Data Availability

Data are available on reasonable request.
