# Supplementary material for: Epidemiology of early-onset and late-onset invasive infections in Australian neonates and infants: a retrospective multicentre study
Source: BMJ Public Health. 2025 Oct 13;3(2):e002733. doi: 10.1136/bmjph-2025-002733 (PMC12519648; doi:10.1136/bmjph-2025-002733)
Supplement: online supplemental file 1 [file bmjph-3-2-s001.docx]

**Supplementary Data**

**Supplementary Figure 1**

Flow chart of isolates included in the analysis

Blood or CSF cultures= 1,165

Organisms in Blood or CSF = 1,286

Two or more likely contaminants reported on the same blood or CSF culture*:

Blood or CSF cultures = 69

Organisms in Blood or CSF = 142

Blood or CSF cultures = 1,100

Organisms in Blood or CSF = 1,144

Bacteria noted on microscopy and not confirmed on culture or via automated method or negative culture

Blood or CSF cultures = 18

Organisms in Blood or CSF = 18

Blood or CSF cultures = 1,086

Organisms in Blood or CSF = 1,126

Repeat cultures within 14 days

Blood or CSF cultures = 112

Organisms in Blood or CSF = 114

Blood or CSF cultures = 979

Organisms in Blood or CSF = 1,012

Duplicate organism and AST from a different blood or CSF culture on the same day

Blood or CSF cultures = 8

Blood or CSF cultures = 971

Organisms in Blood or CSF = 1, 012

\

Coagulase negative staphylococcus reported Day 0-2 of life OR any infant outside a NICU ward

Blood or CSF cultures = 269

Organisms in Blood or CSF = 269

Blood or CSF cultures = 706†

Organisms in Blood or CSF = 743

CSF: Cerebrospinal fluid, NICU: Neonatal Intensive Care Unit or Special Care Nursery

* Contaminants excluded: *Actinomyces naeslundii, Actinomyces odontolyticus, Aerococcus viridans, Bacillus cereus, Bacillus* spp. *Bacteroides vulgatus,* Coagulase negative staphylococcus (CoNS; in infants not admitted to PICU or <72h) *, Corynebacterium minutissimum, Corynebacterium striatum, Empedobacter brevis, Enterococcus durans, Enterococcus hirae, Kocuria varians, Lactococcus lactis subspecies lactis, Leuconostoc pseudomesenteroides, Micrococcus luteus, Micrococcus* spp.*, Moraxella osloensis, Morganella morganii, Neisseria cinerea, Paenibacillus* spp*, Paenibacillus urinalis, Raoultella ornithinolytica, Staphylococcus auricularis, Staphylococcus capitis, Staphylococcus caprae, Staphylococcus epidermidis, Staphylococcus haemolyticus, Staphylococcus hominis, Staphylococcus lugdunensis, Staphylococcus saprophyticus, Staphylococcus spp., Staphylococcus warneri, Staphylococcus xylosus, Streptococcus anginosus, Streptococcus constellatus subspecies pharynges, Streptococcus milleri group, Streptococcus mitis/ Streptococcus oralis* group*, Streptococcus parasanguinis, Streptococcus peroris, Streptococcus salivarius, Streptococcus sanguinis, Streptococcus vestibularis, Viridans group Streptococcus.*

^Further likely contaminants removed (CoNS removed as indicated in the flowchart):*Actinomyces naeslundii, Bacillus cereus , Bacillus species, Corynebacterium minutissimum , Corynebacterium striatum, Empedobacter brevis, Enterococcus durans , Enterococcus hirae, Kocuria varians , Lactococcus lactis subspecies lactis, Leuconostoc pseudomesenteroides, Micrococcus luteus, Micrococcus* spp.*, Paenibacillus* spp., *Paenibacillus urinalis, Raoultella ornithinolytica, Streptococcus anginosus, Streptococcus milleri* group*, Streptococcus mitis/ Streptococcus oralis* group*, Streptococcus parasanguinis, Streptococcus peroris, Streptococcus salivarius, Streptococcus sanguinis, Streptococcus vestibularis, Viridans group Streptococcus.*

† One duplicate (same day) blood culture result retained for additional antimicrobial susceptibility results only.

**Supplementary Table 1:**

**Pre-defined bacteria-antibiotic susceptibility profiles evaluated**

| Gram-positive organisms | |
| --- | --- |
| ***Staphylococcus aureus*** | |
| flu/dicloxacillin | |
| benzylpenicillin | |
| clindamycin | |
| cotrimoxazole | |
| vancomycin | |
| **Streptococcus Group A, B, C and G** | |
| benzylpenicillin | |
| ceftriaxone/cefotaxime | |
| clindamycin | |
| erythromycin | |
| ***Streptococcus pneumoniae*** | |
| benzylpenicillin: blood culture: S / I or DS / R | |
| benzylpenicillin: CSF culture: S / I or DS / R | |
| clindamycin | |
| co-trimoxazole | |
| ceftriaxone/cefotaxime: Blood culture: S / I or DS / R | |
| ceftriaxone/cefotaxime: CSF culture: S / I or DS / R | |
| vancomycin | |
| **Coagulase negative *Staphylococcus* spp.** | |
| flu/dicloxacillin | |
| benzylpenicilin | |
| clindamycin | |
| vancomycin | |
| **Other Gram-positive organisms not otherwise specified** | |
| benzylpenicillin | |
| aminopenicillin | |
| ceftriaxone/cefotaxime | |
| meropenem | |
| vancomycin | |
| clindamycin | |
| co-trimoxazole | |
| Gram-negative pathogens | |
| ***Escherichia coli*** | |
| aminopenicillin | |
| amoxicillin-clavulanate | |
| cephalexin |  |
| ceftriaxone |  |
| cefotaxime |  |
| ceftazidime | |
| cefepime |  |
| meropenem | |
| co-trimoxazole | |
| gentamicin |  |
| amikacin |  |
| tigecycline |  |
| fosfomycin |  |
| ciprofloxacin | |
| ***Klebsiella pneumoniae*** | |
| ceftriaxone |  |
| cefotaxime |  |
| cefepime |  |
| ceftazidime | |
| ciprofloxacin | |
| gentamicin |  |
| amikacin |  |
| co-trimoxazole | |
| fosfomycin |  |
| meropenem | |
| norfloxacin |  |
| tigecycline |  |
| amoxicillin-clavulanate | |
| ***Pseudomonas aeruginosa*** | |
| amikacin |  |
| gentamicin |  |
| meropenem | |
| fosfomycin |  |
| piperacillin-tazobactam | |
| ciprofloxacin | |
| ceftazidime | |
| cefepime |  |
| ***Acinetobacter* spp.** | |
| gentamicin |  |
| amikacin |  |
| meropenem | |
| tigecycline |  |
| minocycline | |
| cefepime |  |
| ciprofloxacin | |
| co-trimoxazole | |
| piperacillin-tazobactam | |
| **Other Gram-negative pathogens not otherwise listed** | |
| aminopenicillin | |
| ampicillin-clavulanate | |
| cephalexin |  |
| ceftriaxone/cefotaxime | |
| ceftazidime | |
| cefepime |  |
| meropenem | |
| co-trimoxazole | |
| trimethoprim | |
| gentamicin |  |
| amikacin |  |
| tigecycline |  |
| fosfomycin |  |
| ciprofloxacin | |
| piperacillin-tazobactam | |

**Supplementary Table 2:**

**Early-onset neonatal sepsis incidence rates across the study period**

**for the five hospitals included in the study**

| Year | Total births in study hospitals | Total EOS cases | EOS incidence per 1,000 livebirths |
| --- | --- | --- | --- |
| 2010 | 9152 | 6 | 0.66 |
| 2011 | NA | 6 | NA |
| 2012 | 9683 | 6 | 0.62 |
| 2013 | 9717 | 9 | 0.93 |
| 2014 | 10106 | 8 | 0.79 |
| 2015 | 14211 | 14 | 0.99 |
| 2016 | 14752 | 11 | 0.75 |
| 2017 | 14616 | 13 | 0.89 |
| 2018 | 14200 | 9 | 0.63 |
| 2019 | 14369 | 11 | 0.77 |

NA: Data not available.

**Supplementary Table 3**

**Gram-positive Blood Culture Isolate Antimicrobial Susceptibility Profiles**

| **Organism** | **Antibiotic** | **Overall** | | **Sites^** | **Year 2010- 2014** | | | | | **Year 2015-2019** | | | | | **p value** | |
| --- | --- | --- | --- | --- | --- | --- | --- | --- | --- | --- | --- | --- | --- | --- | --- | --- |
|  |  | **S/T** | **%S** |  | **n** | **S/T** | **% S** | **95 % CI** | **n** | | **S/T** | **% S** | **95 % CI** |  | |  |
| *Streptococcus agalactiae*, n=76 | Ceftriaxone/Cefotaxime | 11/11 | 100 | All | 19 | 4/4 | 100 | 40-100 | 57 | | 7/7 | 100 | 59-100 |  | |  |
|  |  |  |  | West | 19 | 4/4 | 100 | 40-100 | 0 | | - | - | - |  | |  |
|  | Clindamycin | 27/39 | 69 | All | 19 | 2/4 | 50 | 7-93 | 57 | | 25/35 | 71 | 54-85 | 0.573 | |  |
|  |  |  |  | West | 19 | 2/4 | 50 | 7-93 | 25 | | 11/15 | 73 | 45-92 | 0.557 | |  |
|  | Erythromycin | 32/44 | 73 | All | 19 | 14/19 | 74 | 49-91 | 57 | | 18/25 | 72 | 51-88 | 0.901 | |  |
|  |  |  |  | West | 19 | 14/19 | 74 | 49-91 | 25 | | 18/25 | 72 | 51-88 | 0.901 | |  |
|  | Penicillin | 76/76 | 100 | All | 19 | 19/19 | 100 | 82-100 | 57 | | 57/57 | 100 | 94-100 |  | |  |
|  |  |  |  | West | 19 | 19/19 | 100 | 82-100 | 25 | | 25/25 | 100 | 86-100 |  | |  |
| *Enterococcus faecalis*, n=56 | Ampicillin | 39/39 | 100 | All | 19 | 19/19 | 100 | 82-100 | 37 | | 20/20 | 100 | 83-100 | n/a | |  |
|  |  |  |  | West | 19 | 19/19 | 100 | 82-100 | 20 | | 20/20 | 100 | 83-100 |  | |  |
|  | Clindamycin | 0/13 | 0 | All | 19 | 0/7 | 0 | 0-41 | 37 | | 0/6 | 0 | 0-46 | n/a | |  |
|  |  |  |  | West | 19 | 0/7 | 0 | 0-41 | 20 | | 0/6 | 0 | 0-46 |  | |  |
|  | Cotrimoxazole | 1/16 | 6 | All | 19 | 0/7 | 0 | 0-41 | 37 | | 1/9 | 11 | 0-48 | 1 | |  |
|  |  |  |  | West | 19 | 0/7 | 0 | 0-41 | 20 | | 1/9 | 11 | 0-48 | 1 | |  |
|  | Penicillin | 34/35 | 97 | All | 19 | 17/18 | 94 | 73-100 | 37 | | 17/17 | 100 | 80-100 | 1 | |  |
|  |  |  |  | West | 19 | 17/18 | 94 | 73-100 | 20 | | 17/17 | 100 | 80-100 | 1 | |  |
|  | Vancomycin | 55/55 | 100 | All | 19 | 19/19 | 100 | 82-100 | 37 | | 36/36 | 100 | 90-100 | n/a | |  |
|  |  |  |  | West | 19 | 19/19 | 100 | 82-100 | 20 | | 19/19 | 100 | 82-100 |  | |  |
| *Staphylococcus aureus*, n=53 | Clindamycin | 42/46 | 91 | All | 22 | 22/22 | 100 | 85-100 | 31 | | 20/24 | 83 | 63-95 | 0.11 | |  |
|  |  |  |  | West | 22 | 22/22 | 100 | 85-100 | 9 | | 7/9 | 78 | 40-97 | 0.077 | |  |
|  | Cotrimoxazole | 25/27 | 93 | All | 22 | 16/17 | 94 | 71-100 | 31 | | 9/10 | 90 | 55-100 | 1 | |  |
|  |  |  |  | West | 22 | 16/17 | 94 | 71-100 | 9 | | 9/9 | 100 | 66-100 | 1 | |  |
|  | Flucloxacillin/Dicloxacillin | 44/53 | 83 | All | 22 | 17/22 | 77 | 55-92 | 31 | | 27/31 | 87 | 70-96 | 0.463 | |  |
|  |  |  |  | West | 22 | 17/22 | 77 | 55-92 | 9 | | 8/9 | 89 | 52-100 | 0.642 | |  |
|  | Penicillin | 5/53 | 9 | All | 22 | 2/22 | 9 | 1-29 | 31 | | 3/31 | 10 | 2-26 | 1 | |  |
|  |  |  |  | West | 22 | 2/22 | 9 | 1-29 | 9 | | 0/9 | 0 | 0-34 | 1 | |  |
|  | Vancomycin | 40/40 | 100 | All | 22 | 22/22 | 100 | 85-100 | 31 | | 18/18 | 100 | 81-100 |  | |  |
|  |  |  |  | West | 22 | 22/22 | 100 | 85-100 | 9 | | 9/9 | 100 | 66-100 |  | |  |
| *Streptococcus pneumoniae,* n=4 | Ceftriaxone/Cefotaxime | 1/1 | 100 | All | 0 | - | - | - | 4 | | 1/1 | 100 | 3-100 |  | |  |
|  | Clindamycin | 1/1 | 100 | All | 0 | - | - | - | 4 | | 1/1 | 100 | 3-100 |  | |  |
|  | Penicillin^†^ | 3/4 | 75 | All | 0 | - | - | - | 4 | | 3/4 | 75 | 19-99 |  | |  |
|  | Vancomycin | 1/1 | 100 | All | 0 | - | - | - | 4 | | 1/1 | 100 | 3-100 |  | |  |
| *Streptococcus pyogenes*, n=3 | Ceftriaxone/Cefotaxime | 2/2 | 100 | All | 0 | - | - | - | 2 | | 2/2 | 100 | 16-100 |  | |  |
|  |  |  |  | West | 0 | - | - | - | 2 | | 2/2 | 100 | 16-100 |  | |  |
|  | Erythromycin | 3/3 | 100 | All | 1 | 1/1 | 100 | 3-100 | 2 | | 2/2 | 100 | 16-100 |  | |  |
|  |  |  |  | West | 1 | 1/1 | 100 | 3-100 | 2 | | 2/2 | 100 | 16-100 |  | |  |
|  | Penicillin | 3/3 | 100 | All | 1 | 1/1 | 100 | 3-100 | 2 | | 2/2 | 100 | 16-100 |  | |  |
|  |  |  |  | West | 1 | 1/1 | 100 | 3-100 | 2 | | 2/2 | 100 | 16-100 |  | |  |
| *Actinomyces odontolyticus,* n=2 | Clindamycin | 2/2 | 100 | All | 0 | - | - | - | 2 | | 2/2 | 100 | 16-100 |  | |  |
|  | Cotrimoxazole | 1/1 | 100 | All | 0 | - | - | - | 2 | | 1/1 | 100 | 3-100 |  | |  |
|  | Penicillin | 2/2 | 100 | All | 0 | - | - | - | 2 | | 2/2 | 100 | 16-100 |  | |  |
|  | Vancomycin | 1/1 | 100 | All | 0 | - | - | - | 2 | | 1/1 | 100 | 3-100 |  | |  |
| *Streptococcus cristatus*, n=1 | Ceftriaxone/Cefotaxime | 1/1 | 100 | All | 0 | - | - | - | 1 | | 1/1 | 100 | 3-100 |  | |  |
|  | Penicillin | 1/1 | 100 | All | 0 | - | - | - | 1 | | 1/1 | 100 | 3-100 |  | |  |
|  | Vancomycin | 1/1 | 100 | All | 0 | - | - | - | 1 | | 1/1 | 100 | 3-100 |  | |  |

n: number of blood cultures collected; T: number of isolates tested; S: Susceptible; CI: Confidence interval.

‘West;: Data from New South Wales Health Pathology West Hospitals: Nepean, Children’s Hospital at Westmead and Wagga Wagga Base Hospitals

† 0/4 Streptococcus pneumoniae isolates were resistant to penicillin, 3/4 reported as susceptible and 1/4 “less susceptible”

**Supplementary Table 4**

**Gram-negative** **Blood Culture Antimicrobial Susceptibility Profiles**

| **Organism** | **Antibiotic** | **Overall** | | **Site^** | **Year 2010- 2014** | | | | **Year 2015-2019** | | | | **P value** |
| --- | --- | --- | --- | --- | --- | --- | --- | --- | --- | --- | --- | --- | --- |
|  |  | **S/T** | **% S** |  | **n** | **S/T** | **% S** | **95 % CI** | **n** | **S/T** | **% S** | **95 % CI** |  |
| *Escherichia coli*, n=113 | Amikacin | 75/75 | 100 | All | 42 | 40/40 | 100 | 91-100 | 71 | 35/35 | 100 | 90-100 | - |
|  |  |  |  | West | 42 | 40/40 | 100 | 91-100 | 34 | 34/34 | 100 | 90-100 | - |
|  | Amoxicillin-clavulanate | 59/77 | 77 | All | 42 | 32/40 | 80 | 64-91 | 71 | 27/37 | 73 | 56-86 | 0.47 |
|  |  |  |  | West | 42 | 32/40 | 80 | 64-91 | 34 | 26/34 | 76 | 59-89 | 0.71 |
|  | Ampicillin | 33/74 | 45 | All | 42 | 18/41 | 44 | 28-60 | 71 | 15/33 | 45 | 28-64 | 0.89 |
|  |  |  |  | West | 42 | 18/41 | 44 | 28-60 | 34 | 15/33 | 45 | 28-64 | 0.89 |
|  | Cefepime | 57/65 | 88 | All | 42 | 28/29 | 97 | 82-100 | 71 | 29/36 | 81 | 64-92 | 0.066 |
|  |  |  |  | West | 42 | 28/29 | 97 | 82-100 | 34 | 29/34 | 85 | 69-95 | 0.205 |
|  | Ceftazidime | 68/74 | 92 | All | 42 | 39/40 | 98 | 87-100 | 71 | 29/34 | 85 | 69-95 | 0.088 |
|  |  |  |  | West | 42 | 39/40 | 98 | 87-100 | 34 | 29/34 | 85 | 69-95 | 0.088 |
|  | Ceftriaxone/Cefotaxime | 100/109 | 92 | All | 42 | 38/40 | 95 | 83-99 | 71 | 62/69 | 90 | 80-96 | 0.481 |
|  |  |  |  | West | 42 | 38/40 | 95 | 83-99 | 34 | 29/34 | 85 | 69-95 | 0.236 |
|  | Ciprofloxacin | 73/86 | 85 | All | 42 | 37/41 | 90 | 77-97 | 71 | 36/45 | 80 | 65-90 | 0.185 |
|  |  |  |  | West | 42 | 37/41 | 90 | 77-97 | 34 | 28/34 | 82 | 65-93 | 0.497 |
|  | Trimethoprim-sulfamethoxazole | 55/73 | 75 | All | 42 | 33/39 | 85 | 69-94 | 71 | 22/34 | 65 | 46-80 | 0.049 |
|  |  |  |  | West | 42 | 33/39 | 85 | 69-94 | 34 | 22/32 | 69 | 50-84 | 0.11 |
|  | Gentamicin/Tobramycin | 101/113 | 89 | All | 42 | 40/42 | 95 | 84-99 | 71 | 61/71 | 86 | 76-93 | 0.205 |
|  |  |  |  | West | 42 | 40/42 | 95 | 84-99 | 34 | 27/34 | 79 | 62-91 | 0.069 |
|  | Meropenem | 78/78 | 100 | All | 42 | 41/41 | 100 | 91-100 | 71 | 37/37 | 100 | 91-100 | - |
|  |  |  |  | West | 42 | 41/41 | 100 | 91-100 | 34 | 34/34 | 100 | 90-100 | - |
|  | Tigecycline | 4/4 | 100 | All | 0 | - | - | - | 71 | 4/4 | 100 | 40-100 | - |
|  |  |  |  | West | 0 | - | - | - | 34 | 4/4 | 100 | 40-100 | - |
| *Enterobacter cloacae* complex, n=23 | Amikacin | 16/16 | 100 | All | 12 | 12/12 | 100 | 74-100 | 11 | 4/4 | 100 | 40-100 | - |
|  |  |  |  | West | 12 | 12/12 | 100 | 74-100 | 3 | 3/3 | 100 | 29-100 | - |
|  | Amoxicillin-clavulanate | 0/15 | 0 | All | 12 | 0/12 | 0 | 0-26 | 11 | 0/3 | 0 | 0-71 | - |
|  |  |  |  | West | 12 | 0/12 | 0 | 0-26 | 3 | 0/3 | 0 | 0-71 | - |
|  | Ampicillin | 0/15 | 0 | All | 12 | 0/12 | 0 | 0-26 | 11 | 0/3 | 0 | 0-71 | - |
|  |  |  |  | West | 12 | 0/12 | 0 | 0-26 | 3 | 0/3 | 0 | 0-71 | - |
|  | Cefepime | 14/16 | 88 | All | 12 | 8/8 | 100 | 63-100 | 11 | 6/8 | 75 | 35-97 | 0.467 |
|  |  |  |  | West | 12 | 8/8 | 100 | 63-100 | 3 | 3/3 | 100 | 29-100 | - |
|  | Ceftazidime | 11/15 | 73 | All | 12 | 8/12 | 67 | 35-90 | 11 | 3/3 | 100 | 29-100 | 0.516 |
|  |  |  |  | West | 12 | 8/12 | 67 | 35-90 | 3 | 3/3 | 100 | 29-100 | 0.516 |
|  | Ceftriaxone/Cefotaxime | 2/22 | 9 | All | 12 | 2/12 | 17 | 2-48 | 11 | 0/10 | 0 | 0-31 | 0.480 |
|  |  |  |  | West | 12 | 2/12 | 17 | 2-48 | 3 | 0/3 | 0 | 0-71 | 1 |
|  | Ciprofloxacin | 19/19 | 100 | All | 12 | 12/12 | 100 | 74-100 | 11 | 7/7 | 100 | 59-100 | - |
|  |  |  |  | West | 12 | 12/12 | 100 | 74-100 | 3 | 3/3 | 100 | 29-100 | - |
|  | Trimethoprim-sulfamethoxazole | 17/17 | 100 | All | 12 | 12/12 | 100 | 74-100 | 11 | 5/5 | 100 | 48-100 | - |
|  |  |  |  | West | 12 | 12/12 | 100 | 74-100 | 3 | 3/3 | 100 | 29-100 | - |
|  | Gentamicin/Tobramycin | 20/23 | 87 | All | 12 | 11/12 | 92 | 62-100 | 11 | 9/11 | 82 | 48-98 | 0.59 |
|  |  |  |  | West | 12 | 11/12 | 92 | 62-100 | 3 | 3/3 | 100 | 29-100 | 1 |
|  | Meropenem | 19/20 | 95 | All | 12 | 11/11 | 100 | 72-100 | 11 | 8/9 | 89 | 52-100 | 0.45 |
|  |  |  |  | West | 12 | 11/11 | 100 | 72-100 | 3 | 3/3 | 100 | 29-100 | - |
|  | Piperacillin-tazobactam | 7/17 | 41 | All | 12 | 5/7 | 71 | 29-96 | 11 | 2/10 | 20 | 3-56 | 0.058 |
|  |  |  |  | West | 12 | 5/7 | 71 | 29-96 | 3 | 2/3 | 67 | 9-99 | 1 |
|  | Tigecycline | 1/1 | 100 | All | 0 | - | - | - | 11 | 1/1 | 100 | 3-100 | - |
|  | Trimethoprim | 14/14 | 100 | All | 12 | 11/11 | 100 | 72-100 | 11 | 3/3 | 100 | 29-100 | - |
|  |  |  |  | West | 12 | 11/11 | 100 | 72-100 | 3 | 3/3 | 100 | 29-100 | - |
| *Klebsiella pneumoniae,* n=19 | Amikacin | 8/8 | 100 | All | 6 | 6/6 | 100 | 54-100 | 13 | 2/2 | 100 | 16-100 | - |
|  |  |  |  | West | 6 | 6/6 | 100 | 54-100 | 2 | 2/2 | 100 | 16-100 | - |
|  | Amoxicillin-clavulanate | 10/10 | 100 | All | 6 | 6/6 | 100 | 54-100 | 13 | 4/4 | 100 | 40-100 | - |
|  |  |  |  | West | 6 | 6/6 | 100 | 54-100 | 2 | 2/2 | 100 | 16-100 | - |
|  | Cefepime | 10/10 | 100 | All | 6 | 6/6 | 100 | 54-100 | 13 | 4/4 | 100 | 40-100 | - |
|  |  |  |  | West | 6 | 6/6 | 100 | 54-100 | 2 | 2/2 | 100 | 16-100 | - |
|  | Ceftazidime | 8/8 | 100 | All | 6 | 6/6 | 100 | 54-100 | 13 | 2/2 | 100 | 16-100 | - |
|  |  |  |  | West | 6 | 6/6 | 100 | 54-100 | 2 | 2/2 | 100 | 16-100 | - |
|  | Ceftriaxone/Cefotaxime | 19/19 | 100 | All | 6 | 6/6 | 100 | 54-100 | 13 | 13/13 | 100 | 75-100 | - |
|  |  |  |  | West | 6 | 6/6 | 100 | 54-100 | 2 | 2/2 | 100 | 16-100 | - |
|  | Ciprofloxacin | 9/9 | 100 | All | 6 | 6/6 | 100 | 54-100 | 13 | 3/3 | 100 | 29-100 | - |
|  |  |  |  | West | 6 | 6/6 | 100 | 54-100 | 2 | 2/2 | 100 | 16-100 | - |
|  | Trimethoprim-sulfamethoxazole | 8/8 | 100 | All | 6 | 6/6 | 100 | 54-100 | 13 | 2/2 | 100 | 16-100 | - |
|  |  |  |  | West | 6 | 6/6 | 100 | 54-100 | 2 | 2/2 | 100 | 16-100 | - |
|  | Gentamicin/Tobramycin | 19/19 | 100 | All | 6 | 6/6 | 100 | 54-100 | 13 | 13/13 | 100 | 75-100 | - |
|  |  |  |  | West | 6 | 6/6 | 100 | 54-100 | 2 | 2/2 | 100 | 16-100 | - |
|  | Meropenem | 8/8 | 100 | All | 6 | 6/6 | 100 | 54-100 | 13 | 2/2 | 100 | 16-100 | - |
|  |  |  |  | West | 6 | 6/6 | 100 | 54-100 | 2 | 2/2 | 100 | 16-100 | - |
|  | Norfloxacin | 4/4 | 100 | All | 6 | 2/2 | 100 | 16-100 | 13 | 2/2 | 100 | 16-100 | - |
|  |  |  |  | West | 6 | 2/2 | 100 | 16-100 | 2 | 2/2 | 100 | 16-100 | - |
| *Pseudomonas aeruginosa,* n=14 | Amikacin | 5/5 | 100 | All | 1 | 1/1 | 100 | 3-100 | 13 | 4/4 | 100 | 40-100 | - |
|  |  |  |  | West | 1 | 1/1 | 100 | 3-100 | 4 | 4/4 | 100 | 40-100 | - |
|  | Cefepime | 7/7 | 100 | All | 1 | 1/1 | 100 | 3-100 | 13 | 6/6 | 100 | 54-100 | - |
|  |  |  |  | West | 1 | 1/1 | 100 | 3-100 | 4 | 4/4 | 100 | 40-100 | - |
|  | Ceftazidime | 13/14 | 93 | All | 1 | 0/1 | 0 | 0-98 | 13 | 13/13 | 100 | 75-100 | 0.071 |
|  |  |  |  | West | 1 | 0/1 | 0 | 0-98 | 4 | 4/4 | 100 | 40-100 | 0.2 |
|  | Ciprofloxacin | 7/7 | 100 | All | 1 | 1/1 | 100 | 3-100 | 13 | 6/6 | 100 | 54-100 | - |
|  |  |  |  | West | 1 | 1/1 | 100 | 3-100 | 4 | 4/4 | 100 | 40-100 | - |
|  | Gentamicin/Tobramycin | 14/14 | 100 | All | 1 | 1/1 | 100 | 3-100 | 13 | 13/13 | 100 | 75-100 | - |
|  |  |  |  | West | 1 | 1/1 | 100 | 3-100 | 4 | 4/4 | 100 | 40-100 | - |
|  | Meropenem | 7/7 | 100 | All | 1 | 1/1 | 100 | 3-100 | 13 | 6/6 | 100 | 54-100 | - |
|  |  |  |  | West | 1 | 1/1 | 100 | 3-100 | 4 | 4/4 | 100 | 40-100 | - |
|  | Piperacillin-tazobactam | 14/14 | 100 | All | 1 | 1/1 | 100 | 3-100 | 13 | 13/13 | 100 | 75-100 | - |
|  |  |  |  | West | 1 | 1/1 | 100 | 3-100 | 4 | 4/4 | 100 | 40-100 | - |
| *Acinetobacter spp.,* n=11 | Amikacin | 8/8 | 100 | All | 4 | 4/4 | 100 | 40-100 | 7 | 4/4 | 100 | 40-100 | - |
|  |  |  |  | West | 4 | 4/4 | 100 | 40-100 | 5 | 4/4 | 100 | 40-100 | - |
|  | Cefepime | 6/6 | 100 | All | 4 | 4/4 | 100 | 40-100 | 7 | 2/2 | 100 | 16-100 | - |
|  |  |  |  | West | 4 | 4/4 | 100 | 40-100 | 5 | 2/2 | 100 | 16-100 | - |
|  | Ciprofloxacin | 10/10 | 100 | All | 4 | 4/4 | 100 | 40-100 | 7 | 6/6 | 100 | 54-100 | - |
|  |  |  |  | West | 4 | 4/4 | 100 | 40-100 | 5 | 5/5 | 100 | 48-100 | - |
|  | Trimethoprim-sulfamethoxazole | 4/5 | 80 | All | 4 | 2/3 | 67 | 9-99 | 7 | 2/2 | 100 | 16-100 | 1 |
|  |  |  |  | West | 4 | 2/3 | 67 | 9-99 | 5 | 2/2 | 100 | 16-100 | 1 |
|  | Gentamicin/Tobramycin | 10/11 | 91 | All | 4 | 3/4 | 75 | 19-99 | 7 | 7/7 | 100 | 59-100 | 0.36 |
|  |  |  |  | West | 4 | 3/4 | 75 | 19-99 | 5 | 5/5 | 100 | 48-100 | 0.44 |
|  | Meropenem | 9/9 | 100 | All | 4 | 3/3 | 100 | 29-100 | 7 | 6/6 | 100 | 54-100 | - |
|  |  |  |  | West | 4 | 3/3 | 100 | 29-100 | 5 | 5/5 | 100 | 48-100 | - |
|  | Piperacillin-tazobactam | 10/10 | 100 | All | 4 | 3/3 | 100 | 29-100 | 7 | 7/7 | 100 | 59-100 | - |
|  |  |  |  | West | 4 | 3/3 | 100 | 29-100 | 5 | 5/5 | 100 | 48-100 | - |
| *Serratia marcescens*, n=8 | Amikacin | 3/3 | 100 | All | 2 | 2/2 | 100 | 16-100 | 6 | 1/1 | 100 | 3-100 | - |
|  |  |  |  | West | 2 | 2/2 | 100 | 16-100 | 1 | 1/1 | 100 | 3-100 | - |
|  | Amoxicillin-clavulanate | 0/3 | 0 | All | 2 | 0/2 | 0 | 0-84 | 6 | 0/1 | 0 | 0-98 | - |
|  |  |  |  | West | 2 | 0/2 | 0 | 0-84 | 1 | 0/1 | 0 | 0-98 | - |
|  | Ampicillin | 0/3 | 0 | All | 2 | 0/2 | 0 | 0-84 | 6 | 0/1 | 0 | 0-98 | - |
|  |  |  |  | West | 2 | 0/2 | 0 | 0-84 | 1 | 0/1 | 0 | 0-98 | - |
|  | Cefepime | 4/4 | 100 | All | 2 | 2/2 | 100 | 16-100 | 6 | 2/2 | 100 | 16-100 | - |
|  |  |  |  | West | 2 | 2/2 | 100 | 16-100 | 1 | 1/1 | 100 | 3-100 | - |
|  | Ceftazidime | 3/3 | 100 | All | 2 | 2/2 | 100 | 16-100 | 6 | 1/1 | 100 | 3-100 | - |
|  |  |  |  | West | 2 | 2/2 | 100 | 16-100 | 1 | 1/1 | 100 | 3-100 | - |
|  | Ceftriaxone/Cefotaxime | 1/8 | 12 | All | 2 | 1/2 | 50 | 1-99 | 6 | 0/6 | 0 | 0-46 | 0.25 |
|  |  |  |  | West | 2 | 1/2 | 50 | 1-99 | 1 | 0/1 | 0 | 0-98 | 1 |
|  | Ciprofloxacin | 6/6 | 100 | All | 2 | 2/2 | 100 | 16-100 | 6 | 4/4 | 100 | 40-100 | - |
|  |  |  |  | West | 2 | 2/2 | 100 | 16-100 | 1 | 1/1 | 100 | 3-100 | - |
|  | Trimethoprim-sulfamethoxazole | 3/3 | 100 | All | 2 | 2/2 | 100 | 16-100 | 6 | 1/1 | 100 | 3-100 | - |
|  |  |  |  | West | 2 | 2/2 | 100 | 16-100 | 1 | 1/1 | 100 | 3-100 | - |
|  | Gentamicin/Tobramycin | 8/8 | 100 | All | 2 | 2/2 | 100 | 16-100 | 6 | 6/6 | 100 | 54-100 | - |
|  |  |  |  | West | 2 | 2/2 | 100 | 16-100 | 1 | 1/1 | 100 | 3-100 | - |
|  | Meropenem | 4/4 | 100 | All | 2 | 2/2 | 100 | 16-100 | 6 | 2/2 | 100 | 16-100 | - |
|  |  |  |  | West | 2 | 2/2 | 100 | 16-100 | 1 | 1/1 | 100 | 3-100 | - |
|  | Piperacillin-tazobactam | 5/5 | 100 | All | 2 | 2/2 | 100 | 16-100 | 6 | 3/3 | 100 | 29-100 | - |
|  |  |  |  | West | 2 | 2/2 | 100 | 16-100 | 1 | 1/1 | 100 | 3-100 | - |
|  | Trimethoprim | 3/3 | 100 | All | 2 | 2/2 | 100 | 16-100 | 6 | 1/1 | 100 | 3-100 | - |
|  |  |  |  | West | 2 | 2/2 | 100 | 16-100 | 1 | 1/1 | 100 | 3-100 | - |
| *Klebsiella aerogenes,* n=7 | Amikacin | 2/2 | 100 | All | 0 | - | - | - | 7 | 2/2 | 100 | 16-100 | - |
|  |  |  |  | West | 0 | - | - | - | 2 | 2/2 | 100 | 16-100 | - |
|  | Amoxicillin-clavulanate | 0/2 | 0 | All | 0 | - | - | - | 7 | 0/2 | 0 | 0-84 | - |
|  |  |  |  | West | 0 | - | - | - | 2 | 0/2 | 0 | 0-84 | - |
|  | Ampicillin | 0/2 | 0 | All | 0 | - | - | - | 7 | 0/2 | 0 | 0-84 | - |
|  |  |  |  | West | 0 | - | - | - | 2 | 0/2 | 0 | 0-84 | - |
|  | Cefepime | 3/3 | 100 | All | 0 | - | - | - | 7 | 3/3 | 100 | 29-100 | - |
|  |  |  |  | West | 0 | - | - | - | 2 | 2/2 | 100 | 16-100 | - |
|  | Ceftazidime | 0/2 | 0 | All | 0 | - | - | - | 7 | 0/2 | 0 | 0-84 | - |
|  |  |  |  | West | 0 | - | - | - | 2 | 0/2 | 0 | 0-84 | - |
|  | Ceftriaxone/Cefotaxime | 0/7 | 0 | All | 0 | - | - | - | 7 | 0/7 | 0 | 0-41 | - |
|  |  |  |  | West | 0 | - | - | - | 2 | 0/2 | 0 | 0-84 | - |
|  | Ciprofloxacin | 3/3 | 100 | All | 0 | - | - | - | 7 | 3/3 | 100 | 29-100 | - |
|  |  |  |  | West | 0 | - | - | - | 2 | 2/2 | 100 | 16-100 | - |
|  | Trimethoprim-sulfamethoxazole | 2/2 | 100 | All | 0 | - | - | - | 7 | 2/2 | 100 | 16-100 | - |
|  |  |  |  | West | 0 | - | - | - | 2 | 2/2 | 100 | 16-100 | - |
|  | Gentamicin/Tobramycin | 7/7 | 100 | All | 0 | - | - | - | 7 | 7/7 | 100 | 59-100 | - |
|  |  |  |  | West | 0 | - | - | - | 2 | 2/2 | 100 | 16-100 | - |
|  | Meropenem | 6/6 | 100 | All | 0 | - | - | - | 7 | 6/6 | 100 | 54-100 | - |
|  |  |  |  | West | 0 | - | - | - | 2 | 2/2 | 100 | 16-100 | - |
|  | Trimethoprim | 1/1 | 100 | All | 0 | - | - | - | 7 | 1/1 | 100 | 3-100 | - |
|  |  |  |  | West | 0 | - | - | - | 2 | 1/1 | 100 | 3-100 | - |
| *Klebsiella oxytoca,* n=4 | Cefepime | 2/2 | 100 | All | 0 | - | - | - | 4 | 2/2 | 100 | 16-100 | - |
|  | Ceftriaxone/Cefotaxime | 4/4 | 100 | All | 0 | - | - | - | 4 | 4/4 | 100 | 40-100 | - |
|  | Ciprofloxacin | 1/1 | 100 | All | 0 | - | - | - | 4 | 1/1 | 100 | 3-100 | - |
|  | Gentamicin/Tobramycin | 4/4 | 100 | All | 0 | - | - | - | 4 | 4/4 | 100 | 40-100 | - |
|  | Piperacillin-tazobactam | 3/3 | 100 | All | 0 | - | - | - | 4 | 3/3 | 100 | 29-100 | - |
| *Citrobacter freundii,* n=2 | Amikacin | 1/1 | 100 | All | 0 | - | - | - | 2 | 1/1 | 100 | 3-100 | - |
|  |  |  |  | West | 0 | - | - | - | 1 | 1/1 | 100 | 3-100 | - |
|  | Amoxicillin-clavulanate | 0/1 | 0 | All | 0 | - | - | - | 2 | 0/1 | 0 | 0-98 | - |
|  |  |  |  | West | 0 | - | - | - | 1 | 0/1 | 0 | 0-98 | - |
|  | Ampicillin | 0/1 | 0 | All | 0 | - | - | - | 2 | 0/1 | 0 | 0-98 | - |
|  |  |  |  | West | 0 | - | - | - | 1 | 0/1 | 0 | 0-98 | - |
|  | Cefepime | 2/2 | 100 | All | 0 | - | - | - | 2 | 2/2 | 100 | 16-100 | - |
|  |  |  |  | West | 0 | - | - | - | 1 | 1/1 | 100 | 3-100 | - |
|  | Ceftazidime | 1/1 | 100 | All | 0 | - | - | - | 2 | 1/1 | 100 | 3-100 | - |
|  |  |  |  | West | 0 | - | - | - | 1 | 1/1 | 100 | 3-100 | - |
|  | Ceftriaxone/Cefotaxime | 0/2 | 0 | All | 0 | - | - | - | 2 | 0/2 | 0 | 0-84 | - |
|  |  |  |  | West | 0 | - | - | - | 1 | 0/1 | 0 | 0-98 | - |
|  | Ciprofloxacin | 2/2 | 100 | All | 0 | - | - | - | 2 | 2/2 | 100 | 16-100 | - |
|  |  |  |  | West | 0 | - | - | - | 1 | 1/1 | 100 | 3-100 | - |
|  | Trimethoprim-sulfamethoxazole | 2/2 | 100 | All | 0 | - | - | - | 2 | 2/2 | 100 | 16-100 | - |
|  |  |  |  | West | 0 | - | - | - | 1 | 1/1 | 100 | 3-100 | - |
|  | Gentamicin/Tobramycin | 2/2 | 100 | All | 0 | - | - | - | 2 | 2/2 | 100 | 16-100 | - |
|  |  |  |  | West | 0 | - | - | - | 1 | 1/1 | 100 | 3-100 | - |
|  | Meropenem | 2/2 | 100 | All | 0 | - | - | - | 2 | 2/2 | 100 | 16-100 | - |
|  |  |  |  | West | 0 | - | - | - | 1 | 1/1 | 100 | 3-100 | - |
|  | Piperacillin-tazobactam | 1/2 | 50 | All | 0 | - | - | - | 2 | 1/2 | 50 | 1-99 | - |
|  |  |  |  | West | 0 | - | - | - | 1 | 1/1 | 100 | 3-100 | - |
|  | Trimethoprim | 1/1 | 100 | All | 0 | - | - | - | 2 | 1/1 | 100 | 3-100 | - |
|  |  |  |  | West | 0 | - | - | - | 1 | 1/1 | 100 | 3-100 | - |
| *Pseudomonas oryzihabitans*, n=2 | Amikacin | 2/2 | 100 | All | 1 | 1/1 | 100 | 3-100 | 1 | 1/1 | 100 | 3-100 | - |
|  |  |  |  | West | 1 | 1/1 | 100 | 3-100 | 1 | 1/1 | 100 | 3-100 | - |
|  | Ampicillin | 0/1 | 0 | All | 1 | 0/1 | 0 | 0-98 | 0 | - | - | - | - |
|  |  |  |  | West | 1 | 0/1 | 0 | 0-98 | 0 | - | - | - | - |
|  | Ceftazidime | 1/1 | 100 | All | 0 | - | - | - | 1 | 1/1 | 100 | 3-100 | - |
|  |  |  |  | West | 0 | - | - | - | 1 | 1/1 | 100 | 3-100 | - |
|  | Ceftriaxone/Cefotaxime | 1/1 | 100 | All | 0 | - | - | - | 1 | 1/1 | 100 | 3-100 | - |
|  |  |  |  | West | 0 | - | - | - | 1 | 1/1 | 100 | 3-100 | - |
|  | Ciprofloxacin | 2/2 | 100 | All | 1 | 1/1 | 100 | 3-100 | 1 | 1/1 | 100 | 3-100 | - |
|  |  |  |  | West | 1 | 1/1 | 100 | 3-100 | 1 | 1/1 | 100 | 3-100 | - |
|  | Gentamicin/Tobramycin | 2/2 | 100 | All | 1 | 1/1 | 100 | 3-100 | 1 | 1/1 | 100 | 3-100 | - |
|  |  |  |  | West | 1 | 1/1 | 100 | 3-100 | 1 | 1/1 | 100 | 3-100 | - |
|  | Meropenem | 2/2 | 100 | All | 1 | 1/1 | 100 | 3-100 | 1 | 1/1 | 100 | 3-100 | - |
|  |  |  |  | West | 1 | 1/1 | 100 | 3-100 | 1 | 1/1 | 100 | 3-100 | - |
|  | Piperacillin-tazobactam | 1/1 | 100 | All | 0 | - | - | - | 1 | 1/1 | 100 | 3-100 | - |
|  |  |  |  | West | 0 | - | - | - | 1 | 1/1 | 100 | 3-100 | - |
| *Burkholderia cepacia*, n=1 | Ciprofloxacin | 1/1 | 100 | All | 0 | - | - | - | 1 | 1/1 | 100 | 3-100 | - |
|  | Gentamicin/Tobramycin | 1/1 | 100 | All | 0 | - | - | - | 1 | 1/1 | 100 | 3-100 | - |
| *Haemophilus influenzae*, n=1 | Ceftriaxone/Cefotaxime | 1/1 | 100 | All | 0 | - | - | - | 1 | 1/1 | 100 | 3-100 | - |
| *Haemophilus parainfluenzae*, n=1 | Ceftriaxone/Cefotaxime | 1/1 | 100 | All | 0 | - | - | - | 1 | 1/1 | 100 | 3-100 | - |
| *Leclercia adecarboxylata*, n=1 | Ceftriaxone/Cefotaxime | 1/1 | 100 | All | 0 | - | - | - | 1 | 1/1 | 100 | 3-100 | - |
|  | Ciprofloxacin | 1/1 | 100 | All | 0 | - | - | - | 1 | 1/1 | 100 | 3-100 | - |
|  | Gentamicin/Tobramycin | 1/1 | 100 | All | 0 | - | - | - | 1 | 1/1 | 100 | 3-100 | - |
| *Morganella morganii,* n=1 | Amikacin | 1/1 | 100 | All | 0 | - | - | - | 1 | 1/1 | 100 | 3-100 | - |
|  |  |  |  | West | 0 | - | - | - | 1 | 1/1 | 100 | 3-100 | - |
|  | Amoxicillin-clavulanate | 0/1 | 0 | All | 0 | - | - | - | 1 | 0/1 | 0 | 0-98 | - |
|  |  |  |  | West | 0 | - | - | - | 1 | 0/1 | 0 | 0-98 | - |
|  | Ampicillin | 0/1 | 0 | All | 0 | - | - | - | 1 | 0/1 | 0 | 0-98 | - |
|  |  |  |  | West | 0 | - | - | - | 1 | 0/1 | 0 | 0-98 | - |
|  | Cefepime | 1/1 | 100 | All | 0 | - | - | - | 1 | 1/1 | 100 | 3-100 | - |
|  |  |  |  | West | 0 | - | - | - | 1 | 1/1 | 100 | 3-100 | - |
|  | Ceftazidime | 1/1 | 100 | All | 0 | - | - | - | 1 | 1/1 | 100 | 3-100 | - |
|  |  |  |  | West | 0 | - | - | - | 1 | 1/1 | 100 | 3-100 | - |
|  | Ceftriaxone/Cefotaxime | 0/1 | 0 | All | 0 | - | - | - | 1 | 0/1 | 0 | 0-98 | - |
|  |  |  |  | West | 0 | - | - | - | 1 | 0/1 | 0 | 0-98 | - |
|  | Ciprofloxacin | 1/1 | 100 | All | 0 | - | - | - | 1 | 1/1 | 100 | 3-100 | - |
|  |  |  |  | West | 0 | - | - | - | 1 | 1/1 | 100 | 3-100 | - |
|  | Trimethoprim-sulfamethoxazole | 1/1 | 100 | All | 0 | - | - | - | 1 | 1/1 | 100 | 3-100 | - |
|  |  |  |  | West | 0 | - | - | - | 1 | 1/1 | 100 | 3-100 | - |
|  | Gentamicin/Tobramycin | 1/1 | 100 | All | 0 | - | - | - | 1 | 1/1 | 100 | 3-100 | - |
|  |  |  |  | West | 0 | - | - | - | 1 | 1/1 | 100 | 3-100 | - |
|  | Meropenem | 1/1 | 100 | All | 0 | - | - | - | 1 | 1/1 | 100 | 3-100 | - |
|  |  |  |  | West | 0 | - | - | - | 1 | 1/1 | 100 | 3-100 | - |
|  | Piperacillin-tazobactam | 1/1 | 100 | All | 0 | - | - | - | 1 | 1/1 | 100 | 3-100 | - |
|  |  |  |  | West | 0 | - | - | - | 1 | 1/1 | 100 | 3-100 | - |
|  | Trimethoprim | 1/1 | 100 | All | 0 | - | - | - | 1 | 1/1 | 100 | 3-100 | - |
|  |  |  |  | West | 0 | - | - | - | 1 | 1/1 | 100 | 3-100 | - |
| *Proteus mirabilis,* n=1 | Amikacin | 1/1 | 100 | All | 1 | 1/1 | 100 | 3-100 | 0 | - | - | - | - |
|  |  |  |  | West | 1 | 1/1 | 100 | 3-100 | 0 | - | - | - | - |
|  | Amoxicillin-clavulanate | 1/1 | 100 | All | 1 | 1/1 | 100 | 3-100 | 0 | - | - | - | - |
|  |  |  |  | West | 1 | 1/1 | 100 | 3-100 | 0 | - | - | - | - |
|  | Ampicillin | 1/1 | 100 | All | 1 | 1/1 | 100 | 3-100 | 0 | - | - | - | - |
|  |  |  |  | West | 1 | 1/1 | 100 | 3-100 | 0 | - | - | - | - |
|  | Cefepime | 1/1 | 100 | All | 1 | 1/1 | 100 | 3-100 | 0 | - | - | - | - |
|  |  |  |  | West | 1 | 1/1 | 100 | 3-100 | 0 | - | - | - | - |
|  | Ceftazidime | 1/1 | 100 | All | 1 | 1/1 | 100 | 3-100 | 0 | - | - | - | - |
|  |  |  |  | West | 1 | 1/1 | 100 | 3-100 | 0 | - | - | - | - |
|  | Ceftriaxone/Cefotaxime | 1/1 | 100 | All | 1 | 1/1 | 100 | 3-100 | 0 | - | - | - | - |
|  |  |  |  | West | 1 | 1/1 | 100 | 3-100 | 0 | - | - | - | - |
|  | Ciprofloxacin | 1/1 | 100 | All | 1 | 1/1 | 100 | 3-100 | 0 | - | - | - | - |
|  |  |  |  | West | 1 | 1/1 | 100 | 3-100 | 0 | - | - | - | - |
|  | Trimethoprim-sulfamethoxazole | 1/1 | 100 | All | 1 | 1/1 | 100 | 3-100 | 0 | - | - | - | - |
|  |  |  |  | West | 1 | 1/1 | 100 | 3-100 | 0 | - | - | - | - |
|  | Gentamicin/Tobramycin | 1/1 | 100 | All | 1 | 1/1 | 100 | 3-100 | 0 | - | - | - | - |
|  |  |  |  | West | 1 | 1/1 | 100 | 3-100 | 0 | - | - | - | - |
|  | Meropenem | 1/1 | 100 | All | 1 | 1/1 | 100 | 3-100 | 0 | - | - | - | - |
|  |  |  |  | West | 1 | 1/1 | 100 | 3-100 | 0 | - | - | - | - |
|  | Piperacillin-tazobactam | 1/1 | 100 | All | 1 | 1/1 | 100 | 3-100 | 0 | - | - | - | - |
|  |  |  |  | West | 1 | 1/1 | 100 | 3-100 | 0 | - | - | - | - |
|  | Tigecycline | 0/1 | 0 | All | 1 | 0/1 | 0 | 0-98 | 0 | - | - | - | - |
|  |  |  |  | West | 1 | 0/1 | 0 | 0-98 | 0 | - | - | - | - |
|  | Trimethoprim | 1/1 | 100 | All | 1 | 1/1 | 100 | 3-100 | 0 | - | - | - | - |
|  |  |  |  | West | 1 | 1/1 | 100 | 3-100 | 0 | - | - | - | - |

S: Susceptible; T: number of isolates tested; West: New South Wales Health Pathology West hospitals; n: number of blood isolates; CI: Confidence interval

^ Data available for New South Wales Health Pathology West hospitals (Nepean hospital, Children’s Hospital at Westmead, and Wagga Wagga Base Hospital) only

**Supplementary Table 5.**

**Gram-negative Cerebrospinal Fluid Culture Antimicrobial Susceptibility Profiles**

| **Pathogen** | **Antibiotic**  (number susceptible/number tested; % susceptible) | | | | | | | | | | |
| --- | --- | --- | --- | --- | --- | --- | --- | --- | --- | --- | --- |
|  | **Ampicillin** | **AMX-CLAV** | **Ceftriaxone** | **Cefepime** | **Ceftazidime** | **TMP-SMX** | **Ciprofloxacin** | **Gentamicin** | **Amikacin** | **PIP-TAZ** | **Meropenem** |
| *Escherichia coli,* n=7 | 1/2 (50) | 2/2 (100) | 6/6 (100) | 2/2 (100) | 2/2 (100) | 2/3 (67) | 4/4 (100) | 6/6(100) | 2/2 (100) |  | 3/3 (100) |
| *Enterobacter cloacae* complex n=2 |  |  | 0/2 (0) | 2/2 (100) |  |  |  | 2/2 (100) |  | 0/1 (0) |  |
| *Pseudomonas aeruginosa*, n=2 |  |  |  |  | 2/2 (100) |  | 1/1 (100) | 2/2 (100) |  | 1/1 (100) | 1/1 (100) |
| *Pseudomonas luteola,* n=1 |  |  |  |  | 1/1 (100) |  | 1/1 (100) | 1/1 (100) |  |  |  |
| *Klebsiella oxytoca,* n=1 |  |  | 1/1 (100) |  |  |  | 1/1 (100) | 1/1 (100) |  |  |  |
| *Neisseria meningitidis,* n=1 |  |  | 1/1 (100) |  |  |  |  |  |  |  |  |
| *Serratia liquefaciens,* n=1 |  |  | 1/1 (100) |  |  |  | 1/1 (100) | 1/1 (100) |  |  |  |
| *Serratia marcescens,* n=1 |  |  | 0/1 (0) |  |  |  | 1/1 (100) | 1/1 (100) |  |  |  |

AMX-CLAV: Amoxicillin-clavulanate; Ceftriaxone: Ceftriaxone or Cefotaxime; TMP-SMX: Trimethoprim-sulfamethoxazole; Gentamicin: Gentamicin or Tobramycin; PIP-TAZ: Piperacillin-tazobactam

**Supplementary Table 6**

**Gram-positive CSF isolate antimicrobial susceptibility profiles**

| **Pathogen** | **Antibiotic**  number susceptible/number tested (% susceptible) | | | | | | |
| --- | --- | --- | --- | --- | --- | --- | --- |
|  | **Penicillin** | **Erythromycin** | **Ceftriaxone** | **Flucloxacillin** | **Clindamycin** | **TMP-SMX** | **Vancomycin** |
| *Streptococcus agalactiae*, n=11 | 11/11 (100) | 0/4 (0) | 5/5 (100) |  | 1/2 (50) |  |  |
| *Staphylococcus aureus*, n=5 | 2/5 (40) |  |  | 4/5 (80) | 4/4 (100) | 1/2 (50) | 2/2 (100) |
| *Enterococcus faecalis*, n=1 |  |  |  |  |  |  | 1/1 (100) |

Ceftriaxone: Ceftriaxone or Cefotaxime, Flucloxacillin: Flucloxacillin or Dicloxacillin; TMP-SMX: Trimethoprim-sulfamethoxazole
